# Interpretable Machine Learning Identification of Dietary and Metabolic Factors for Metabolic Syndrome in Southern China: A Cross-Sectional Study

**DOI:** 10.3390/nu17213368

**Published:** 2025-10-27

**Authors:** Xi Meng, Yiting Fang, Shuaijing Zhang, Panpan Huang, Jian Wen, Jiewen Peng, Xingfen Yang, Guiyuan Ji, Wei Wu

**Affiliations:** 1 NMPA Key Laboratory for Safety Evaluation of Cosmetics, Guangdong Provincial Key Laboratory of Tropical Disease Research, School of Public Health, Southern Medical University, Guangzhou 510515, China; 2 Guangdong Provincial Institute of Public Health, Guangdong Provincial Center for Disease Control and Prevention, Guangzhou 511400, China; 3 Guangdong Provincial Center for Disease Control and Prevention, Guangzhou 511400, China

**Keywords:** metabolic syndrome, dietary, metabolic factors, machine learning, southern China

## Abstract

**Background**: Metabolic syndrome (MetS) is a rising public health concern in Southern China, with limited evidence available on dietary and metabolic factors. This cross-sectional study employed an interpretable machine learning (ML) approach to identify factors that could inform clinical and community interventions. **Methods**: Data were obtained from the Guangdong Nutrition Surveys conducted in 2015 and 2022, including sociodemographic information, lifestyle patterns, physical examinations, laboratory measurements and dietary intake information (collected via repeated 24-h dietary recalls). Potentially relevant variables were selected using the Least Absolute Shrinkage and Selection Operator (LASSO) regression and incorporated into seven ML models. Model performance was primarily assessed using the area under the receiver operating characteristic curve (AUC), and the contribution of identified features was interpreted through SHapley Additive exPlanations (SHAP). **Results**: This analysis included 5593 participants, of whom 1103 were classified as having MetS. After removing collinear features, the ML models retained 19 candidate variables, which were selected through LASSO regression. XGBoost achieved the best performance (AUC: 0.834; F1 score: 0.537) with a misclassification rate of 27.1%. SHAP analysis highlighted body mass index (BMI), age, and uric acid levels as major risk factors, while insoluble dietary fiber, carbohydrate and specific micronutrients exhibited protective associations. **Conclusions**: Machine learning identified key dietary and metabolic factors of MetS. Integrating these factors into clinical practice and public health initiatives may enhance early detection and support personalized prevention strategies for MetS in Southern China.

## 1. Introduction

Metabolic syndrome (MetS), defined by a cluster of metabolic abnormalities, including central obesity, hypertension, hyperglycemia, dyslipidemia, and insulin resistance [1,2], significantly elevates the risk of developing type 2 diabetes [3], cardiovascular diseases [4] and stroke [5]. It has become a major global health challenge, affecting approximately one in four adults worldwide [1,6]. In the U.S., MetS prevalence reached 41.8% during 2017–2018 [7], while in China, it was 31.1% among adults aged 20 years and older in 2015–2017 [8], with Southern China seeing an increase from 21.3% in 2010 [9] to 27.4% in 2015–2017 [8]. This increasing trend highlights the urgent need for effective prevention and management strategies within both clinical and community-based healthcare systems.

In China, particularly in southern provinces such as Guangdong, the rapid economic development and shifts in dietary patterns have contributed to the disease burden of MetS. National initiatives, including “Healthy China 2030”, aim to strengthen MetS management in primary care systems, yet their implementation faces significant challenges. A key limitation is the lack of accurate, individualized risk assessment tools, which hinders the identification of high-risk populations.

Dietary improvement is essential for the prevention and management of MetS [1,10], as it influences metabolic processes such as blood pressure regulation, lipid profiles, glucose metabolism, and body fat distribution. Macronutrients [11,12,13] and micronutrients [14,15,16,17] play critical roles in regulating insulin sensitivity, oxidative stress, and lipid metabolism regulation. However, the impact of diet is significantly modulated by regional variations and the complexity of dietary components. The *Cantonese (Lingnan) dietary pattern*, characterized by high consumption of vegetables, fruits, aquatic products, and light cooking, may offer cardiometabolic benefits, but the optimal nutritional strategy for MetS prevention in this region is remains unclear. Limited research on specific dietary and metabolic factors within this pattern hinders the development of culturally tailored preventive strategies. Traditional analytical methods, such as principal component analysis (PCA) [18], are limited in their ability to capture the complex interactions and nonlinear relationships between diet and metabolism, thereby limiting their ability to support precise population stratification and personalized intervention strategies.

In contrast, machine learning (ML) methods are well-suited to analyzing high-dimensional and complex health data, enabling the detection of nonlinear relationships and variable interactions that are not readily discernible through traditional statistical techniques [19]. This study further incorporates the SHapley Additive exPlanations (SHAP) analysis to enhance model interpretability, thereby transforming the key factors identified by ML into transparent, actionable insights that can support decision-making in real-world healthcare settings.

Building on this foundation, this study aims to systematically identify key dietary and metabolic markers associated with MetS within the dietary patterns of the Lingnan region and to quantify their relative contributions to disease risk. Ultimately, this research seeks to provide primary care institutions in southern China with evidence-based, actionable, and personalized nutrition intervention strategies as well as clinical decision support tools, thereby facilitating the implementation and scalability of precision approaches to the prevention and management of metabolic syndrome in real-world healthcare settings.

## 2. Materials and Methods

### 2.1. Data Source and Study Population

This cross-sectional study was based on data from the China National Nutrition Surveys (CNNS), conducted by the Chinese Center for Disease Control and Prevention in 2015 and 2022. Detailed information on the survey design has been previously published [20]. For this analysis, only data from Guangdong Province were included.

**Figure 1:**
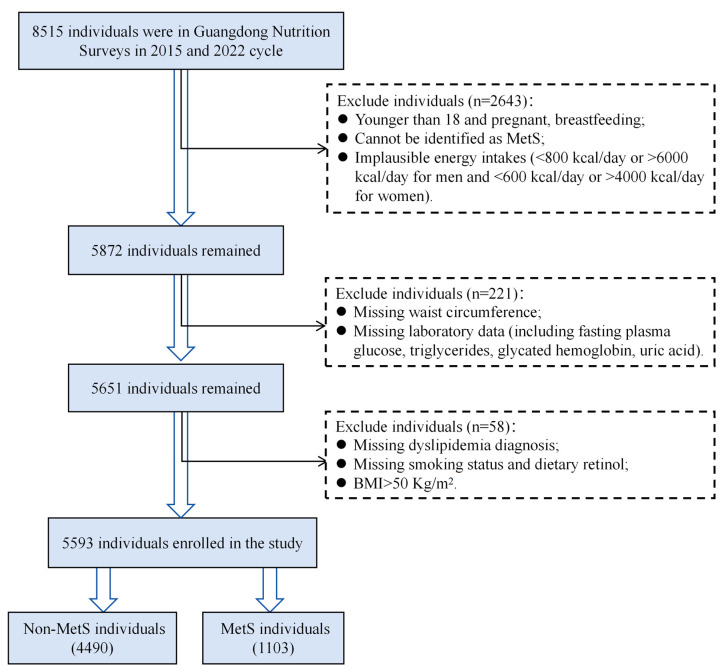
Flowchart of the study participants screening in the study. BMI, body mass index; MetS, metabolic syndrome.

Eligible participants were adults aged 18 years or older with complete demographic, dietary, and biochemical data. Pregnant women, lactating mothers, and individuals with implausible energy intakes (<800 kcal/day or >6000 kcal/day for men; <600 kcal/day or >4000 kcal/day for women) were excluded. A total of 5593 individuals were included in the final analysis. The participant selection flowchart is shown in Figure 1. The survey protocols were approved by the Ethics Committee of the Chinese Center for Disease Control and Prevention (Approval Nos. 201519-B and 2022-008), and all participants provided written informed consent. This study was reported in accordance with TRIPOD (the Transparent Reporting of a Multivariable Prediction Model for Individual Prognosis or Diagnosis) statement [21]. The completed checklist is available in Table S1.

### 2.2. Definition of Metabolic Syndrome

MetS was defined in accordance with the Chinese Guideline for the Prevention and Treatment of Type 2 Diabetes (CDS 2020) [22]. Diagnosis required at least three of the following: (1) abdominal obesity: male waist circumference ≥ 90 cm, female waist circumference ≥ 85 cm; (2) hyperglycemia: fasting plasma glucose (FPG) ≥ 6.1 mmol/L or 2 h postprandial blood glucose ≥7.8 mmol/L and/or prior diagnosis and treatment of diabetes; (3) hypertension: blood pressure ≥ 130/85 mmHg and/or confirmed hypertension under treatment; (4) fasting triglyceride (TG) ≥ 1.70 mmol/L; (5) fasting high-density lipoprotein cholesterol (HDL-C) < 1.04 mmol/L.

### 2.3. Dietary Assessment

Dietary intake was assessed by trained interviewers using a repeated 24 h dietary recall method [23], including one weekend day, to account for day-to-day variability and better estimate usual intake. This method is widely used in large-scale epidemiological studies and national nutrition surveys. To improve accuracy, participants were assisted with food models and standardized portion-size photographs to minimize recall omission and estimation error. Dietary intakes were calculated based on the Chinese Food Composition Table [24]. The average intake over three days was used in all analyses.

### 2.4. Data Preprocessing and Model Development

To minimize redundancy, variables with correlation coefficients exceeding 0.8 were excluded. The dataset was randomly divided into training (70%) and test (30%) sets. Subsequently, variable selection was performed with the Least Absolute Shrinkage and Selection Operator (LASSO) regression, which applies an L1 penalty to shrink uninformative coefficients toward zero, thereby improving model parsimony and mitigating overfitting.

Class imbalance between MetS and non-MetS groups was addressed in the training set using the Synthetic Minority Oversampling Technique (SMOTE) [25], which generates synthetic minority class samples based on nearest-neighbor interpolation.

Seven machine learning algorithms were employed to construct models: Light Gradient Boosting Machine (LightGBM), Random Forest (RF), Extreme Gradient Boosting (XGBoost), Support Vector Machine (SVM), Logistic Regression (LR), K-nearest neighbors (KNN), and Naïve Bayes (NB). These algorithms were chosen for their complementary strengths. LightGBM utilizes gradient-based sampling and feature bundling to efficiently process large datasets. RF aggregates multiple decision trees to enhance robustness and reduce variance. XGBoost extends gradient boosting with regularization mechanisms for flexibility and speed. SVM identifies optimal separating hyperplanes in high-dimensional space via kernel functions. LR offers interpretability and stability when applied to structured data. KNN classifies instances based on similarity to neighboring observations. NB provides computational efficiency for high-dimensional categorical data. All models were trained using 10-fold cross-validation, and hyperparameters were optimized via grid search to maximize performance while avoiding overfitting.

### 2.5. Performance of the ML Models

Performance was evaluated by multiple metrics: accuracy, sensitivity, specificity, positive predictive value (PPV), negative predictive value (NPV), F1 score, and classification error rate. The primary criterion for model selection was the area under the receiver operating characteristic curve (AUC). Confusion matrices were constructed to provide detailed classification outcomes, including true positives, true negatives, false positives, and false negatives.

### 2.6. Interpretability Methods for the Optimal Models

To address the “black-box” nature of ML, the SHAP algorithm was applied to the best-performing model. SHAP assigns each feature a contribution score that reflects its additive effect on model output, enabling both global feature ranking and instance-level interpretability [26].

### 2.7. Other Covariates

Covariates included sociodemographic variables (age, sex [male/female], residence [urban/rural], education level [primary school or below, junior middle school, senior high school or above]), behavioral factors (smoking status [current vs. non-smoker], alcohol consumption [current vs. non-drinker]), and physical activity level (categorized as mild, moderate, or heavy based on occupation).

Physical examinations were conducted after an overnight fast. Measurements included height, weight, waist circumference, and blood pressure. Height and weight were recorded to the nearest 0.1 cm and 0.1 kg, respectively. Blood pressure was measured three times at 1 min intervals using standardized instruments, and the average of the three readings was used in analysis. Body mass index (BMI) was calculated as weight (kg) divided by height squared (m^2^).

Venous blood samples (8 mL) were collected to measure FPG, TG, total cholesterol (TC), low-density lipoprotein cholesterol (LDL-C), HDL-C, glycated hemoglobin (HbA1c), and uric acid (UA). All samples were processed under standardized conditions and analyzed in a central laboratory.

### 2.8. Statistical Analysis

Participant characteristics stratified by MetS status were analyzed using survey-weighted methods. Continuous variables are presented as means ± standard deviations for normally distributed data or medians with interquartile ranges for skewed distributions, while categorical variables are reported as frequencies and percentages. Between-group comparisons were performed using the weighted chi-square test for categorical variables, analysis of variance (ANOVA) for normally distributed continuous variables, and the Kruskal–Wallis *H* test for non-normally distributed data.

All analyses were conducted in R (version 4.5.1) and Python (version 3.13.5) using established statistical packages. A two-tailed *p*-value < 0.05 was considered statistically significant.

## 3. Results

### 3.1. Characteristics of Participants

A total of 5593 participants were included in the analysis, comprising 4490 (80.3%) individuals without MetS and 1103 (19.7%) with MetS. The baseline characteristics of these participants are shown in Table S2. Participants with MetS were older and exhibited higher BMI, waist circumference, and blood pressure levels (all *p* < 0.01). Significant differences were also observed in sex, education, and smoking status. Dietary comparisons indicated that the MetS group had lower intakes of carbohydrate, insoluble dietary fiber, and several micronutrients, including biotin, choline, iron, copper, and manganese (all *p* < 0.05). Biochemical profiles further confirmed the MetS phenotype, showing elevated FPG, HbA1c, TG, and TC as well as reduced HDL-C levels (all *p* < 0.01). Correlations between dietary and non-dietary variables are illustrated in Figure S1.

### 3.2. Variables Selection Using LASSO Regression

**Figure 2:**
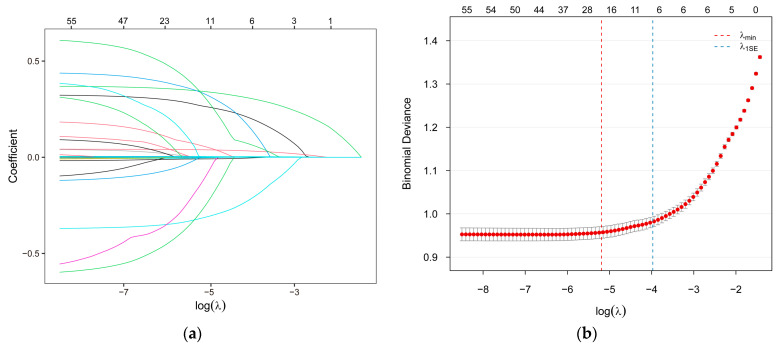
The LASSO regression process for feature selection. (**a**) LASSO path plot; (**b**) LASSO cross-validation error plot. LASSO, the Least Absolute Shrinkage and Selection Operator.

LASSO regression analysis was performed with 10-fold cross-validation and 1000 iterations to identify the most relevant variables. Figure 2a displays the LASSO coefficient paths, illustrating how coefficients converge toward zero with increasing regularization penalty. The accompanying cross-validation error plot (Figure 2b) shows the mean squared error for each λ values, which was used to select the value that minimizes the prediction error. Ultimately, the optimal λ value was determined to be 0.0464 (Figure 2b), yielding 19 covariates for inclusion in the machine learning models. These included seven clinical and demographic variables (age, sex, education, physical activity, BMI, TC, UA) and 12 dietary variables (insoluble dietary fiber, retinol, carbohydrate, pantothenic acid, biotin, folate, choline, vitamin K, potassium, copper, manganese, iodine).

### 3.3. Model Evaluation and Comparison

**Figure 3:**
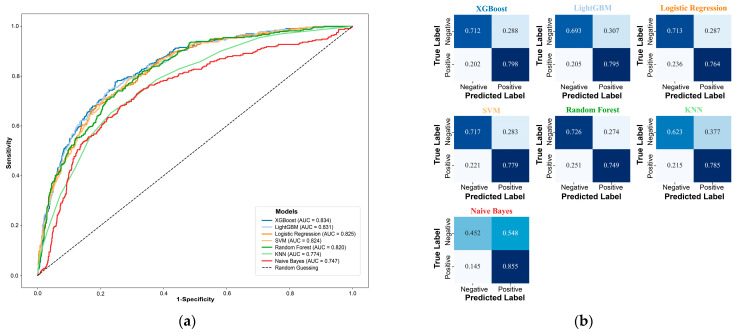
Evaluation the performance of all machine learning models. (**a**) ROC curves; (**b**) Confusion matrices. XGBoost, Extreme Gradient Boosting; LightGBM, Light Gradient Boosting Machine; SVM, Support Vector Machine; KNN, K-nearest neighbors.

Table 1 summarizes the performance metrics of seven machine learning models (XGBoost, LightGBM, LR, SVM, RF, KNN, and NB) on the test set. Among them, the XGBoost model demonstrated superior performance, achieving the highest AUC value (0.834; 95% CI: 0.809–0.856) (Figure S2) and F1 score (0.537). The ROC curve (Figure 3a) indicates that the curve of XGBoost is closest to the upper left corner, demonstrating its outstanding discrimination ability. The confusion matrix (Figure 3b) reveals high sensitivity (0.798) and a high negative predictive value (0.935). Additionally, it maintains a relatively low misclassification rate (27.1%) (Table 1). Consequently, XGBoost model was selected as the optimal model for subsequent interpretability analysis.

### 3.4. Importance of Features Interpretation Using SHAP

**Figure 4:**
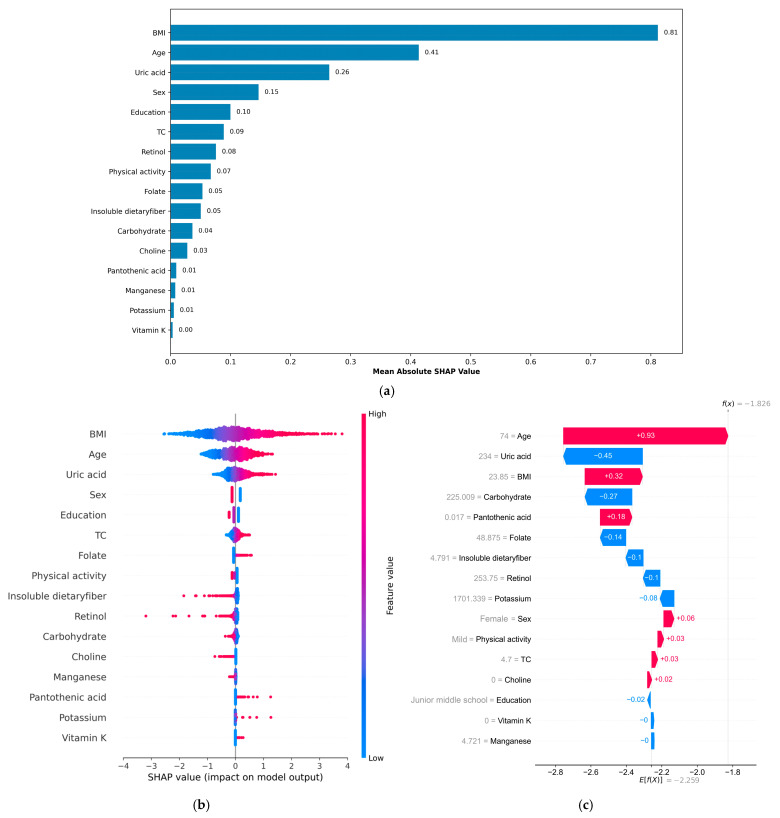
This is a SHAP analysis on the best machine learning model (XGBoost model) to enhance the interpretability of the result. (**a**) SHAP summary bar plot; (**b**) SHAP summary plot; (**c**) SHAP waterfall plot. BMI, body mass index; TC, total cholesterol; SHAP, Shapley Additive exPlanations.

SHAP analysis was applied to interpret the final model’s output. The computation of SHAP values provides a clear and interpretable method for evaluating the importance of individual features in a model. The global explanation provides an overview of the model’s overall functionality. As shown in the SHAP summary bar plot (Figure 4a), feature contributions were ranked by their mean SHAP values in descending order, with BMI, age, and UA identified as the three most influential features in the model. Furthermore, the SHAP summary plot (Figure 4b) visually illustrates the direction and magnitude of each feature’s influence on the model, highlighting BMI, age, and UA as the most robust positive biomarkers. In contrast, higher dietary intakes of retinol, insoluble fiber, carbohydrates, biotin, choline, and manganese were associated with negative SHAP values, indicating potential protective effects against MetS. For individual-level interpretation, specific plots were generated. The SHAP waterfall plot (Figure 4c) for the third participant demonstrates how each feature contributes to the model output for that individual using the XGBoost framework. The actual values of each feature and their corresponding SHAP values in the graph illustrate the extent to which each feature exerts a positive or negative influence on the outcome. For instance, an age of 74 years and a BMI of 23.85 kg/m^2^ contributed significantly to the outcome with positive SHAP values of +0.93 and +0.32, respectively. Conversely, a UA level of 234 μmol/L and a carbohydrate level of 225.009 g/day were associated with negative contributions, yielding SHAP values of −0.45 and −0.27, respectively. Additionally, SHAP dependence plots (Figure S3) were constructed to visualize the individual contributions of the three most influential features—BMI, age, and UA—ranked by mean SHAP values. The plots revealed that higher BMI, older age, and elevated UA levels were consistently associated with increased SHAP values, suggesting that they may have a positive contribution to MetS.

## 4. Discussion

The complex interplay between nutritional and metabolic factors and MetS remains inadequately characterized in the Southern Chinese population. To address this knowledge gap, we employed an interpretable machine learning approach—XGBoost—to identify key factors of MetS by integrating a comprehensive set of nutritional and clinical variables. Our analysis revealed a distinct metabolic and dietary intake that differentiate individuals with and without MetS, underscoring the value of multidimensional data integration for understanding this multifaceted condition.

By leveraging SHAP-based interpretability, BMI, age, and UA were identified as the most prominent risk factors, whereas insoluble dietary fiber, carbohydrate, retinol, biotin, choline, and manganese were associated with protective effects. These findings confirm the multifactorial nature of MetS and highlight the crucial role of dietary composition in its pathogenesis and prevention. The XGBoost model demonstrated robust performance in our study, outperforming logistic regression and other tree-based algorithms. This illustrates its capacity to capturing complex, nonlinear relationships inherent in multidimensional health data.

This study adds to the growing body of evidence supporting the application of interpretable machine learning in nutritional epidemiology. While previous studies—including those by Cai et al. [19] and a South Korean cohort investigation [27]—have demonstrated the robust predictive accuracy of XGBoost for MetS, our work focuses specifically on the Lingnan population, which is distinguished by its distinct dietary culture. More importantly, we integrated SHAP analysis to clarify the direction and magnitude of individual feature contributions. This shift from mere factor identification to model interpretability provides actionable insights into modifiable risk factors for MetS within this specific demographic. Our results reinforce the multifactorial etiology of MetS and emphasize the pivotal role of dietary composition in both its development and prevention.

After identifying the key influencing factors through SHAP analysis, we further interpreted the primary contributors by integrating evidence from the literature with regional characteristics. In this study, BMI emerged as the strongest determinant of MetS, a finding consistent with epidemiological data across diverse populations worldwide, including those in Asia [28,29,30], Europe [31], and the United States [32]. The underlying pathophysiological mechanism are well established: excess adiposity, particularly visceral fat, initiates a cascade of metabolic disturbances, positioning obesity as a central target for MetS prevention and intervention. Although BMI has limitations in capturing fat distribution and does not account for the metabolically obese normal-weight (MONW) phenotype [33,34], its high SHAP value in this study underscores its substantial contribution to metabolic dysregulation within the Lingnan population. Advancing age was another dominant factor, aligning with evidence from global cohorts and population-based studies that demonstrate a marked increase in MetS prevalence with aging [7], reflecting the cumulative burden of metabolic burden across the life course.

Notably, UA ranked as the third most influential factor in our analysis, surpassing several conventional risk factors. This result is supported by robust findings from prospective cohort studies, which consistently report an association between elevated UA levels and increased incidence of MetS [35]. Furthermore, genome-wide association studies and Mendelian randomization analyses suggest a potential causal role of uric acid in cardiovascular and metabolic traits [36]. The high prevalence of hyperuricemia in Guangdong—exceeding 20% [37,38]—indicates that local dietary patterns may amplify this risk. The Lingnan region is rich in poultry, and there is a saying that “no chicken, no feast”. “Boiled Chicken Slices”, “Cantonese Roast Goose” and “Chaozhou Foie Gras” are famous Cantonese delicacies. Moreover, the regional diet features high consumption of poultry (164.8 g/day) and aquatic products (57 g/day), both of which are rich in purines and substantially above the national average. Additionally, the traditional practice of preparing slow-cooked soups results in significant purine leaching in the broth, further promoting uric acid production [39]. Mechanistically, hyperuricemia has been shown to contribute to insulin resistance, visceral adiposity, oxidative stress, and endothelial dysfunction—core pathways implicated in MetS pathogenesis [40]. Thus, in this population, both biological mechanisms and distinctive regional dietary habits converge to reinforce UA as a pivotal determinant of MetS.

Dietary and nutritional factors also played a significant role. Insoluble dietary fiber emerged as a consistent protective factor against MetS, consistent with existing evidence linking higher intake to enhanced cardiometabolic profiles [11,41]. Potential mechanisms include improved satiety, modulation of bile acid metabolism, and beneficial effects on gut microbiota, all of which contribute to improved insulin sensitivity and lipid homeostasis [42,43]. Carbohydrate intake was inversely associated with MetS, a relationship likely driven by carbohydrate quality rather than quantity—particularly the consumption of whole grains and high-fiber foods [44,45,46].

In this study, retinol (vitamin A) exhibited a protective association, although its biological effects are complex and may follow a U-shaped dose–response curve, in which both deficiency and excess intake are linked to increased risk, while moderate intake appears to confer potential benefits [47,48]. Our findings support this protective hypothesis, suggesting that the effects may vary according to baseline nutritional status, intake levels, and genetic predispositions. Other micronutrients—biotin, choline, and manganese—also showed inverse associations with MetS. Biotin functions as a coenzyme in key reactions of energy metabolism [49], while choline, which is abundant in regional staples such as fish and soy products, plays a critical role in lipid metabolism and insulin sensitivity [50,51]. Manganese, primarily derived from rice [52] in this population, acts as a cofactor in antioxidant defense systems and mitochondrial energy pathways [53,54]. Collectively, these findings reflect the characteristics of the Lingnan dietary pattern, which emphasizes whole grains, legumes, nuts, and rice-based staples—foods rich in dietary fiber and essential micronutrients. This coherence strengthens the plausibility of our results and highlights the importance of interpreting nutrient–disease relationships within the context of local dietary practices.

This study has several important strengths. First, to our knowledge, this is the first application of interpretable machine learning methods in southern China to integrate demographic, lifestyle, and nutritional-metabolic factors and construct a multi-dimensional framework for identifying potential determinants of MetS. Second, the use of stratified, multistage sampling to obtain a large, representative sample substantially enhances the robustness and regional applicability of the findings. More importantly, the key factors identified—such as BMI, UA, and specific nutrients—can serve as the foundation for developing stratified risk assessment tools, enabling earlier identification of high-risk individuals beyond conventional diagnostic criteria. For instance, individuals with hyperuricemia may benefit from personalized dietary interventions targeting local high-purine food practices, such as the consumption of traditional slow-cooked soups and certain aquatic products. These insights can be integrated into existing chronic disease management systems, providing a practical framework for the precise prevention and management of MetS through patient education, individualized goal setting, and continuous monitoring of intervention outcomes. Ultimately, this approach is expected to reduce the disease burden of metabolic syndrome in this region.

### Limitations

Several limitations should be acknowledged. First, the cross-sectional design precludes the establishment of temporal sequence or causality between dietary factors and MetS. Consequently, our findings should be interpreted as hypothesis-generating and require future validation, rather than serving as evidence of causal relationships. Second, dietary intake was assessed using 3-day 24-h recalls—a method prone to recall bias and potentially insufficient for capturing long-term habitual eating patterns—thereby increasing the likelihood of non-differential misclassification of exposures. Although this approach represents an improvement over single-day assessments, future studies should employ more robust dietary assessment methods, such as repeated 24-h recalls combined with food frequency questionnaires (FFQs) and objective nutritional biomarkers (e.g., urinary nitrogen, serum carotenoids), improve measurement precision and support stronger causal inference. Third, the LASSO method was applied for feature selection rather than for controlling confounding; thus, residual confounding from unmeasured genetic, lifestyle (e.g., sleep quality, psychological stress), and environmental factors may still affect the observed associations. Furthermore, while SHAP values offer insight into variable importance, they are based on the assumption of feature independence and do not indicate causal relationships. Persistent multicollinearity among predictors, even after preprocessing, may complicate mechanistic interpretation.

To address these limitations, we recommend that future research utilize longitudinal cohort studies and randomized controlled trials to confirm the causal roles of the identified factors. Additionally, integrating multi-omics data (e.g., genomics, metabolomics) with comprehensive lifestyle and environmental assessments will be critical for elucidating the complex determinants of MetS and advancing the development of personalized prevention strategies.

## 5. Conclusions

This study applied an interpretable machine learning framework to identify potential related factors of MetS in a Southern Chinese population. The model highlighted the dominant roles of BMI, age, and UA as risk factors, while underscoring the protective effects of insoluble dietary fiber and several micronutrients. These findings emphasize the multifactorial nature of MetS and the importance of integrating both clinical and nutritional data for risk assessment. The results support the development of stratified prevention strategies, such as personalized dietary guidance targeting local purine-rich foods and promoting fiber-rich diets. Future longitudinal studies are warranted to confirm causality and translate these findings into precise clinical and public health interventions.

## Figures and Tables

**Table 1:** Performance comparison of different machine learning models across multiple evaluation metrics.

Model	AUC	Sensitivity	Specificity	Accuracy	PPV	NPV	F1 Score	Recall	CER
XGBoost	0.834	0.798	0.712	0.729	0.405	0.935	0.537	0.798	0.271
LightGBM	0.831	0.795	0.693	0.713	0.389	0.932	0.522	0.795	0.287
Logistic Regression	0.825	0.764	0.713	0.723	0.395	0.925	0.521	0.764	0.277
SVM	0.824	0.779	0.717	0.729	0.404	0.930	0.532	0.779	0.271
Random Forest	0.820	0.749	0.726	0.731	0.402	0.922	0.523	0.749	0.269
KNN	0.774	0.785	0.623	0.655	0.339	0.922	0.473	0.785	0.345
Naive Bayes	0.747	0.855	0.452	0.532	0.277	0.927	0.419	0.855	0.468

Abbreviations: AUC, the area under the ROC curve; PPV, positive predictive value; NPV, negative predictive value; CER, Classification Error Rate; XGBoost, Extreme Gradient Boosting; LightGBM, Light Gradient Boosting Machine; SVM, Support Vector Machine; KNN, K-nearest neighbors.

## Data Availability

The original contributions presented in the study are included in the article; further inquiries can be directed to the corresponding author. The data are not publicly available according to the National Institute for Nutrition and Health and the Chinese Center for Disease Control and Prevention.

## Supplementary Materials

The following supporting information can be downloaded at https://www.mdpi.com/article/10.3390/nu17213368/s1. Table S1: A complete and transparent report of the TRIPOD checklist for multivariable prediction models for individual prognosis or diagnosis; Table S2: Baseline characteristics of the participants; Figure S1: The correlation coefficients between dietary features and other variables; Figure S2: The AUC Forest plot of model performance selection; Figure S3: The SHAP dependence plot for the top 3 most important features (BMI, age and UA) for the third participant.

## Abbreviations

AUC	the area under the ROC curve
BMI	body mass index
CER	Classification Error Rate
CDS 2020	Chinese Guideline for the Prevention and Treatment of Type 2 Diabetes
CNNS	China National Nutrition Surveys
DBP	diastolic blood pressure
FFQs	food frequency questionnaires
FPG	fasting plasma glucose
HbA1c	glycated hemoglobin
HDL-C	high-density lipoprotein cholesterol
KNN	K-nearest neighbors
LASSO	The Least Absolute Shrinkage and Selection Operator
LDL-C	low-density lipoprotein cholesterol
LightGBM	Light Gradient Boosting Machine
LR	Logistic Regression
MetS	metabolic syndrome
MONW	metabolically obese normal weight
ML	machine learning
NB	Naïve Bayes
NPV	negative predictive value
PCA	principal component analysis
PPV	positive predictive value
RF	Random Forest
SBP	systolic blood pressure
SHAP	Shapley Additive exPlanations
SMOTE	Synthetic Minority Oversampling Technique
SVM	Support Vector Machine
TC	total cholesterol
TG	triglycerides
TRIPOD	Transparent Reporting of a Multivariable Prediction Model for Individual Prognosis or Diagnosis
UA	uric acid
XGBoost	Extreme Gradient Boosting
