# Supplementary material for: Interpretable Machine Learning Identification of Dietary and Metabolic Factors for Metabolic Syndrome in Southern China: A Cross-Sectional Study
Source: Nutrients. 2025 Oct 27;17(21):3368. doi: 10.3390/nu17213368 (PMC12610728; doi:10.3390/nu17213368)
Supplement: Supplementary file 1 [file nutrients-17-03368-s001.zip › nutrients-3914644-supplementary.pdf]

Table S1. A complete and transparent report of the TRIPOD checklist for multivariable prediction models for individual prognosis or diagnosis. One point to note is that our study utilized cross-sectional survey data and employed interpretable machine learning methods to identify potential risk factors for diseases, rather than developing the prediction model itself. Therefore, certain items in the TRIPOD checklist are not directly applicable, and these have been designated as "Not Applicable (NA)." A comprehensive review of our article has been conducted. For applicable items, the relevant content has been highlighted in red font, and the corresponding page and section numbers in the revised manuscript have been documented in the checklist.

Section/Topic			Checklist Item	Page
**Title and abstract**				
Title	1	D; V	Identify the study as developing and/or validating a multivariable prediction model, the target population, and the outcome to be predicted.	Page 1
Abstract	2	D; V	Provide a summary of objectives, study design, setting, participants, sample size, predictors, outcome, statistical analysis, results, and conclusions.	Page 1, Section Abstract
**Introduction**				
Background and objectives	3a	D; V	Explain the medical context (including whether diagnostic or prognostic) and rationale for developing or validating the multivariable prediction model, including references to existing models.	Page 2, Section Introduction
	3b	D; V	Specify the objectives, including whether the study describes the development or validation of the model or both.	NA
**Methods**				
Source of data	4a	D; V	Describe the study design or source of data (e.g., randomized trial, cohort, or registry data), separately for the development and validation data sets, if applicable.	Page 2, Section Method 2.1
	4b	D; V	Specify the key study dates, including start of accrual; end of accrual; and, if applicable, end of follow-up.	NA
Participants	5a	D; V	Specify key elements of the study setting (e.g., primary care, secondary care, general population), including number and location of centres.	Page 2, Section Method 2.1
	5b	D; V	Describe eligibility criteria for participants.	Page 2, Section Method 2.1 and Figure 1
	5c	D; V	Give details of treatments received, if relevant.	NA
Outcome	6a	D; V	Clearly define the outcome that is predicted by the prediction model, including how and when assessed.	Page 3, Section Method 2.2
	6b	D; V	Report any actions to blind assessment of the outcome to be predicted.	Page 3, Section Method 2.4 (Paragraph 1-2)
Predictors	7a	D; V	Clearly define all predictors used in developing or validating the multivariable prediction model, including how and when they were measured.	Page 3-4, Section Method 2.3 and 2.7
	7b	D; V	Report any actions to blind assessment of predictors for the outcome and other predictors.	Page 3, Section Method 2.4 (Paragraph 1: remove correlation coefficients >0.8)
Sample size	8	D; V	Explain how the study size was arrived at.	Page 2, Section Method 2.1 (Reference 20)
Missing data	9	D; V	Describe how missing data were handled (e.g., complete-case analysis, single imputation, multiple imputation) with details of any imputation method.	Page 2, Section Method 2.1 and Figure 1
Statistical analysis methods	0a	D	Describe how predictors were handled in the analyses.	Page 3, Section Method 2.4 (Paragraph 1: LASSO method)
	0b	D	Specify type of model, all model-building procedures (including any predictor selection), and method for internal validation.	Page 3, Section Method 2.4 (Paragraph 3)
	0c	V	For validation, describe how the predictions were calculated.	NA
	0d	D; V	Specify all measures used to assess model performance and, if relevant, to compare multiple models.	Page 3, Section Method 2.5

	0e	V	Describe any model updating (e.g., recalibration) arising from the validation, if done.	NA
Risk groups	11	D; V	Provide details on how risk groups were created, if done.	NA
Development vs. validation	12	V	For validation, identify any differences from the development data in setting, eligibility criteria, outcome, and predictors.	NA
**Results**				
Participants	3a	D; V	Describe the flow of participants through the study, including the number of participants with and without the outcome and, if applicable, a summary of the follow-up time. A diagram may be helpful.	Figure 1
	3b	D; V	Describe the characteristics of the participants (basic demographics, clinical features, available predictors), including the number of participants with missing data for predictors and outcome.	Page 4, Section Results 3.1 and Table 1
	3c	V	For validation, show a comparison with the development data of the distribution of important variables (demographics, predictors and outcome).	NA
Model development	4a	D	Specify the number of participants and outcome events in each analysis.	NA
	4b	D	If done, report the unadjusted association between each candidate predictor and outcome.	NA
Model specification	5a	D	Present the full prediction model to allow predictions for individuals (i.e., all regression coefficients, and model intercept or baseline survival at a given time point).	NA
	5b	D	Explain how to the use the prediction model.	Page 5, Section Results 3.4
Model performance	16	D; V	Report performance measures (with CIs) for the prediction model.	Page 5, Section Results 3.3
Model-updating	17	V	If done, report the results from any model updating (i.e., model specification, model performance).	NA
**Discussion**				
Limitations	18	D; V	Discuss any limitations of the study (such as nonrepresentative sample, few events per predictor, missing data).	Page 12, Section Discussion (Paragraph limitations)
Interpretation	9a	V	For validation, discuss the results with reference to performance in the development data, and any other validation data.	NA
	9b	D; V	Give an overall interpretation of the results, considering objectives, limitations, results from similar studies, and other relevant evidence.	Page10-12, Section Discussion
Implications	20	D; V	Discuss the potential clinical use of the model and implications for future research.	Page 12, Section Discussion (Paragraph 2 and 4)
**Other information**				
Supplementary information	21	D; V	Provide information about the availability of supplementary resources, such as study protocol, Web calculator, and data sets.	NA
Funding	22	D; V	Give the source of funding and the role of the funders for the present study.	Page 13, Section Author Contributions and Funding

Abbreviations: TRIPOD, Transparent Reporting of a multivariable prediction model for Individual Prognosis or Diagnosis; NA, not applicable.

Table S2. Baseline characteristics of the participants.

	Overall (5,593)	Non-MetS (4,490)	MetS (1,103)	*P-value*
**Age, years**	52.24 ± 14.70	51.01 ± 14.82	57.24 ± 13.08	**<0.001**
**Sex (%)**				**<0.001**
Male	2565 (45.9%)	1948 (43.4%)	617 (55.9%)	
Female	3028 (54.1%)	2542 (56.6%)	486 (44.1%)	
**Residence (%)**				0.361
Urban	3838 (68.6%)	3068 (68.3%)	770 (69.8%)	
Rural	1755 (31.4%)	1422 (31.7%)	333 (30.2%)	
**Education (%)**				**<0.001**
Primary school or below	2242 (40.1%)	1726 (38.4%)	516 (46.8%)	
Junior middle school	2482 (44.4%)	2014 (44.9%)	468 (42.4%)	
Senior high school or above	869 (15.5%)	750 (16.7%)	119 (10.8%)	
**Physical activity (%)**				0.369
Mild	3065 (54.8%)	2453 (54.6%)	612 (55.5%)	
Moderate	1153 (20.6%)	916 (20.4%)	237 (21.5%)	
Heavy	1375 (24.6%)	1121 (25.0%)	254 (23.0%)	
**Smoking status (%)**				**<0.001**
Current smoker	4209 (75.3%)	3427 (76.3%)	782 (70.9%)	
Non smoker	1384 (24.7%)	1063 (23.7%)	321 (29.1%)	
**Alcohol consumption (%)**				0.177
Current drinker	3649 (65.2%)	2949 (65.7%)	700 (63.5%)	
Non drinker	1944 (34.8%)	1541 (34.3%)	403 (36.5%)	
**Dyslipidemia (%)**				**<0.001**
Yes	3209 (57.4%)	3009 (67.0%)	200 (18.1%)	
No	2384 (42.6%)	1481 (33.0%)	903 (81.9%)	
**Hypertension (%)**				**<0.001**
Yes	2844 (50.8%)	2709 (60.3%)	135 (12.2%)	
No	2749 (49.2%)	1781 (39.7%)	968 (87.8%)	
**Type 2 diabetes (%)**				**<0.001**
Yes	4691 (83.9%)	4144 (92.3%)	547 (49.6%)	
No	902 (16.1%)	346 (7.7%)	556 (50.4%)	
**BMI, Kg/m^2**	23.40 ± 3.65	22.74 ± 3.27	26.79 ± 3.58	**<0.001**
**Waist circumference, cm**	80.37 ± 10.26	78.19 ± 9.17	91.52 ± 8.09	**<0.001**
**SBP, mmHg**	124.07 ± 18.29	120.89 ± 16.50	140.34 ± 18.40	**<0.001**
**DBP, mmHg**	76.55 ± 10.67	74.75 ± 9.68	85.78 ± 10.77	**<0.001**
**FPG, mmol/L**	5.43 (0.89)	5.19 (0.75)	6.66 (1.82)	**<0.001**
**TG, mmol/L**	1.51 (0.46)	1.22 (0.68)	3.01 (1.41)	**<0.001**
**TC, mmol/L**	5.04 ± 1.03	4.97 ± 0.99	5.36 ± 1.16	**<0.001**
**LDL-C, mmol/L**	3.17 ± 0.92	3.17 ± 0.88	3.19 ± 1.10	0.707
**HDL-C, mmol/L**	1.28 ± 0.35	1.33 ± 0.34	1.02 ± 0.27	**<0.001**
**HbA1c,%**	5.11 ± 0.89	4.97 ± 0.70	5.80 ± 1.31	**<0.001**
**Uric acid,μmol/L**	357.87 ± 98.27	347.98 ± 93.41	408.53 ± 106.62	**<0.001**
**Energy, kcal/day**	1,960.11 ± 714.58	1,967.43 ± 702.57	1,922.60 ± 772.43	0.359
**Protein, g/day**	77.83 (34.96)	77.62 (39.47)	78.93 (48.71)	0.591

**Total fat, g/day**	94.38 ± 47.65	94.32 ± 47.20	94.68 ± 49.93	0.913
**Carbohydrate, g/day**	204.75 ± 94.20	206.98 ± 92.22	193.31 ± 103.05	**0.015**
**Cholesterol, mg/day**	426.99 (319.58)	425.23 (304.31)	436.01 (388.57)	0.609
**Total dietary fiber, g/day**	2.87 (7.03)	2.89 (7.53)	2.75 (3.57)	0.534
**Insoluble dietary fiber, g/day**	8.78 (12.33)	8.97 (12.53)	7.85 (11.22)	**0.041**
**Soluble dietary fiber, g/day**	0.10 (0.55)	0.09 (0.50)	0.14 (0.77)	0.242
**Vitamin A, mcg/day**	606.67 (550.71)	607.95 (545.47)	600.10 (577.01)	0.859
**Retinol, mcg/day**	156.97 (253.57)	158.21 (259.15)	150.60 (222.84)	0.794
**Thiamin, mcg/day**	0.88 ± 0.46	0.89 ± 0.45	0.84 ± 0.50	0.145
**Riboflavin, mg/day**	0.94 (0.62)	0.94 (0.62)	0.94 (0.64)	0.874
**Niacin, mg/day**	19.26 (12.21)	19.28 (12.20)	19.15 (12.23)	0.833
**Pantothenic acid, mg/day**	0.01 (0.07)	0.01 (0.06)	0.01 (0.08)	0.27
**Pyridoxine, mg/day**	0.10 (0.27)	0.10 (0.29)	0.10 (0.14)	0.965
**Biotin, mcg/day**	1.73 (11.55)	1.85 (12.42)	1.06 (5.11)	**0.012**
**Folate, mcg/day**	46.12 (71.58)	43.74 (68.91)	58.32 (82.88)	**0.001**
**Choline, mg/day**	4.25 (14.53)	4.73 (15.58)	1.80 (6.41)	**<0.001**
**Vitamin B12, mcg/day**	0.07 (0.41)	0.07 (0.39)	0.09 (0.52)	0.545
**Carotene, mcg/day**	2,521.27 (2,427.12)	2,517.48 (2,311.82)	2,540.67 (2,949.04)	0.907
**Vitamin C, mg/day**	98.64 (80.32)	98.40 (80.65)	99.89 (78.60)	0.737
**Vitamin D, mcg/day**	3.71 (29.04)	3.97 (30.38)	2.39 (20.84)	0.162
**Vitamin E, mg/day**	26.39 (18.18)	26.67 (18.18)	24.91 (18.13)	0.198
**Vitamin K, mg/day**	3.32 (23.00)	2.70 (18.51)	6.50 (38.39)	0.104
**Calcium, mg/day**	435.55 (299.94)	439.90 (309.35)	413.25 (245.08)	0.063
**Phosphorus, mg/day**	982.44 ± 471.27	983.54 ± 464.51	976.79 ± 504.64	0.793
**Potassium, mg/day**	3,143.65 (1,057.85)	3,118.49 (1,051.93)	3,272.57 (1,091.51)	0.796
**Sodium, mg/day**	5,145.16 (4,555.53)	5,084.35 (4,553.61)	5,456.68 (4,554.82)	0.233
**Magnesium, mg/day**	270.63 (153.67)	271.91 (157.95)	264.08 (129.40)	0.281
**Iron, mg/day**	23.24 (29.23)	23.63 (31.11)	21.21 (16.39)	**0.01**
**Zinc, mg/day**	12.03 (9.28)	11.88 (6.95)	12.83 (16.73)	0.32
**Selenium, mcg/day**	54.97 (36.86)	54.78 (35.53)	55.92 (43.05)	0.614
**Copper, mg/day**	1.75 (1.57)	1.79 (1.48)	1.59 (1.96)	**0.009**
**Manganese, mg/day**	5.08 (9.69)	5.21 (10.46)	4.43 (3.80)	**0.01**
**Iodine, mcg/day**	572.12 (297.69)	572.63 (295.67)	569.52 (307.98)	0.848
**Magnesium, mg/day**	270.63 (153.67)	271.91 (157.95)	264.08 (129.40)	0.281

Abbreviations: BMI, Body mass index; SBP, systolic blood pressure; DBP, diastolic blood pressure; FPG, fasting plasma glucose; TG, triglycerides; TC, total cholesterol; LDL-C, low-density lipoprotein cholesterol; HDL-C, high-density lipoprotein cholesterol; HbA1c, glycated hemoglobin.

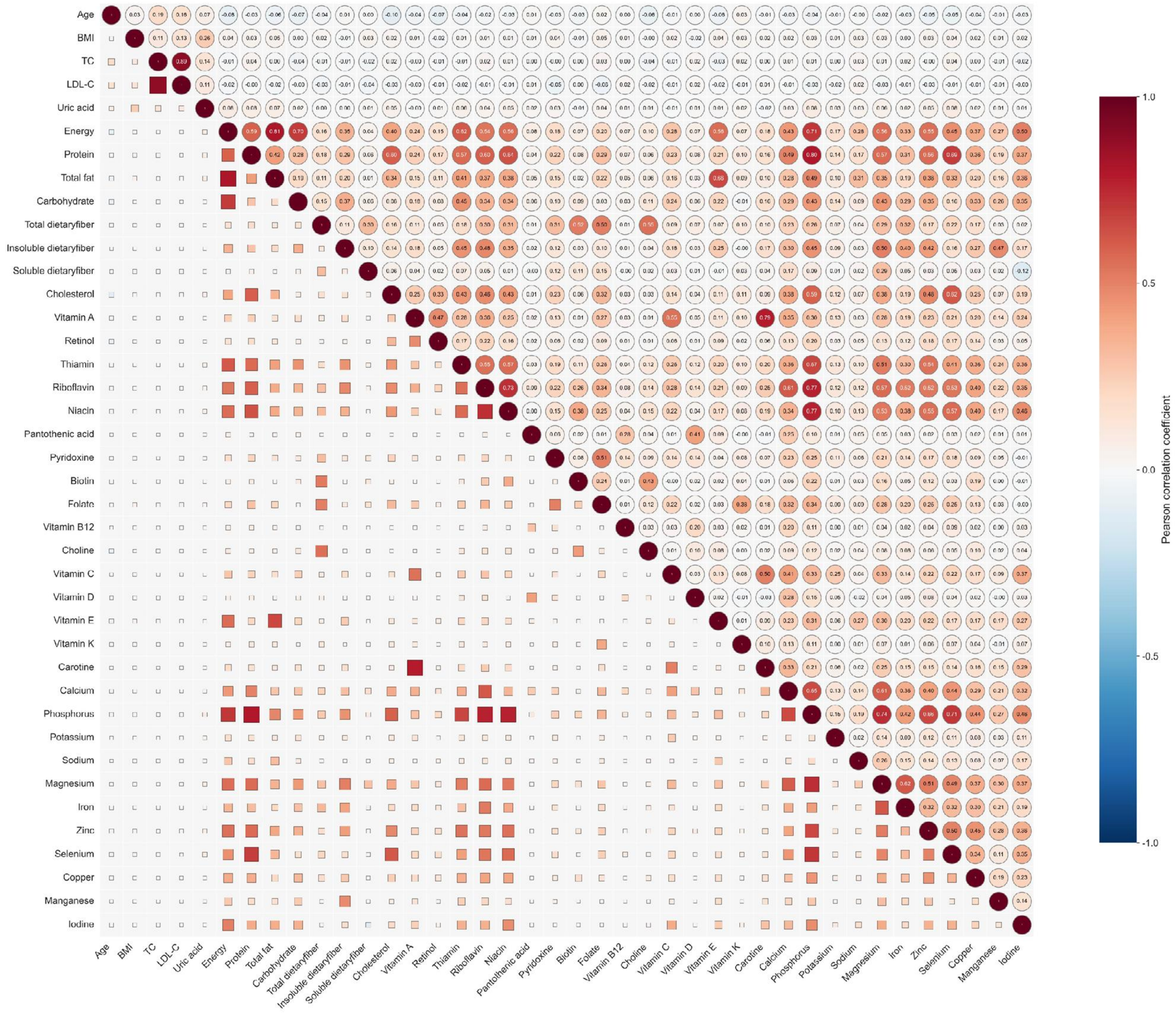

Figure S1. The correlation coefficients between dietary features and other variables. BMI, Body mass index; TC, total cholesterol; LDL-C, low-density lipoprotein cholesterol.

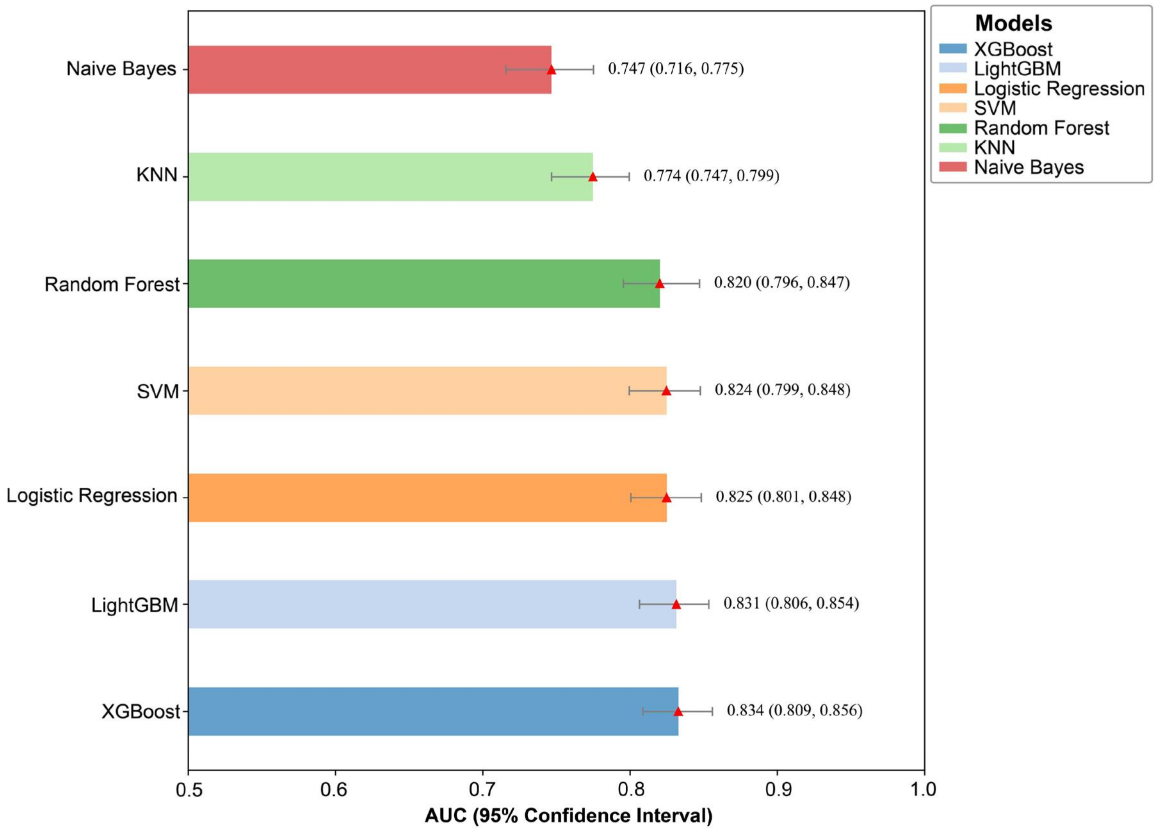

Figure S2. The AUC forest plot of model performance selection. AUC, the area under the ROC curve; XGBoost, Extreme Gradient Boosting; LightGBM, Light Gradient Boosting Machine; SVM, Support Vector Machine; KNN, K-nearest neighbors.

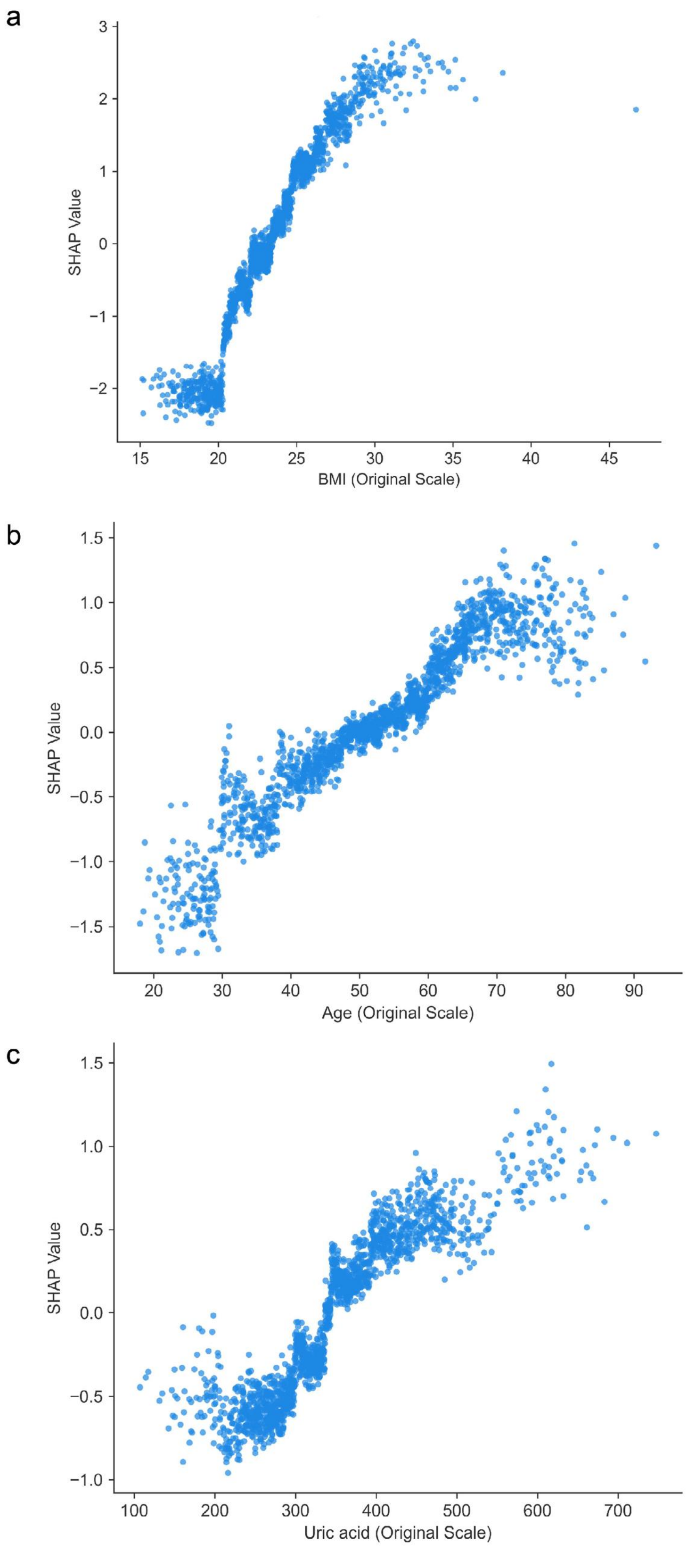

Figure S3. The SHAP dependence plot for the top 3 most important features (BMI, age and UA) for the third participant. The dependence plots revealed that higher BMI, increased age, and elevated uric acid levels were positively correlated with SHAP values. BMI, Body mass index; UA, uric acid.
